# Dataset of plasmid DNA extraction using different magnetic nanoparticles (MNPs)

**DOI:** 10.1016/j.dib.2016.10.013

**Published:** 2016-10-26

**Authors:** H. Rahnama, A. Sattarzadeh, F. Kazemi, N. Ahmadi, F. Sanjarian, Z. Zand

**Affiliations:** a Agricultural Biotechnology Research Institute of Iran (ABRII), Agricultural Research, Education and Extension Organization (AREEO), Karaj, Iran; b Institute for Advanced Studies in Basic Sciences (IASBS), Zanjan, Iran; c National Institute of Genetic Engineering and Biotechnology (NIGEB), Tehran, Iran; d Gil Nanogene Biotech Co., Research and Development Department, Tehran, Iran

**Keywords:** pBI121, Bacteria, Magnetic nanoparticles, Plasmid, TEM, EDS

## Abstract

In this dataset we integrated figures related to bacterial transformation using pBI121 plasmid and complementary analysis for magnetic nanoparticles (MNPs) characterizations. The structural map of pBI121 plasmid was drawn by Vector NTI software using the complete sequence of binary vector pBI121. *Escherichia coli* bacteria transformed using pBI121 plasmid and were grown on the selection media containing kanamycin.

MNPs were characterized by energy dispersive spectroscopy (EDS) and transmission electron microscopy (TEM). Finally, the overall efficiency of different MNPs (Fe_3_O_4_, Fe_3_O_4_/SiO_2_, Fe_3_O_4_/SiO_2_/TiO_2_) in plasmid DNA isolation was compared using gel electrophoresis analysis. The data supplied in this article supports the accompanying publication “Comparative study of three magnetic nano-particles (FeSO_4_, FeSO_4_/SiO_2_, FeSO_4_/SiO_2_/TiO_2_) in plasmid DNA extraction” (H. Rahnama, A. Sattarzadeh, F. Kazemi, N. Ahmadi, F. Sanjarian, Z. Zand, 2016) [1].

**Specifications Table**

**Table:** 

Subject area	Biology
More specific subject area	Nanotechnology in biology
Type of data	Figure
How data was acquired	Vector NTI software v. 11.5, Energy Dispersive X-ray Spectroscopy (EDS, JEM-2100), Transmission Electron Microscopy (TEM) images were recorded on a CM-120 microscope (Philips, 120 kV), Agarose Gel Electrophoresis
Data format	Raw, analyzed
Experimental factors	Three MNPs were used for plasmid extraction
Experimental features	A binary vector pBI121 were transformed in the *Escherichia coli* bacteria. Transformed bacteria were grown on a section media containing kanamycin. The efficiency of three MNPs (Fe_3_O_4_, Fe_3_O_4_/SiO_2_, Fe_3_O_4_/SiO_2_/TiO_2_) in the plasmid DNA extraction was compared at the same conditions.
Data source location	Karaj, Iran
Data accessibility	Data is provided with this article

**Value of the data**

• The data can help in understanding the acquired resistance to kanamycin in *E. coli* bacteria by transformation using pBI121 plasmid.
• The data is important to confirm the presence of different elements in MNP structure.
• The data is useful as it presents the differential efficiency of MNPs in isolation of DNA.

## 1 Data

Fig. 1 represent the physical map of pBI121 binary plasmid used for genetic transformation of *E. coli* bacteria. The bacteria harboring pBI121 were grown as single colonies on LB media containing kanamycine as a selection agent (Fig. 2).

EDS data presented in Fig. 3 show the existence of Ti, Fe, and Si elements on the surface of the magnetic oxide microspheres. Particle size was determined by TEM analysis as described in Fig. 4. DNA recovery of different MNPs (Fe_3_O_4_, Fe_3_O_4_/SiO_2_, Fe_3_O_4_/SiO_2_/TiO_2_) in plasmid DNA isolation was compared in Fig. 5.

## 2 Experimental design, materials and methods

The structural map of pBI121 plasmid was drawn by Vector NTI software v. 11.5 using the complete sequence of the binary vector pBI121 (GenBank: AF485783.1) (Fig. 1).

### 2.1 Bacterial transformation

The plasmid DNA, pBI121 was replicated in the bacterial host cells, DH5α *E. coli*. Five nanograms (5 ng) of pBI121 (Fig. 1) was gently mixed with competent *E. coli* cells [1]. After incubation on ice for 30 min, the bacteria were incubated at 42 °C for 90 s and then kept on ice for 2 min. After heat shock, 1 ml of Luira–Bertani (LB) broth media was added to the tube containing *E. coli* and incubated for 1 h at 37 °C for recovery [2]. Thereafter, 100 μl of *E. coli* culture was spread on the LB agar containing 50 mg/l kanamycin and incubated at 37 °C overnight for the colony formation. *E.coli* harboring pBI121 plasmid appeared as bacterial colonies on LB media containing selection agent kanamycin (Fig. 2).The bacteria cultures were utilized for plasmid extraction experiments.

#### 2.1.1 Characterization of MNPs

The surface morphology of products was analyzed utilizing a transmission electron microscopy (TEM) images were recorded on a CM-120 microscope (Philips, 120 kV) (Fig. 3) and characterized by energy dispersive X-ray spectroscopy (EDS, JEM-2100) (Fig. 4) [3], [4].

#### 2.1.2 Plasmid DNA extraction

DNA recovery efficiency of MNPs (Fe_3_O_4_, Fe_3_O_4_/SiO_2_, Fe_3_O_4_/SiO_2_/TiO_2_) in plasmid DNA isolation is compared in Fig. 5. The isolated DNA by MNPs was separated by electrophoresis on a 1% agarose gel and then visualized under UV light after post staining by Gelred.

## Figures and Tables

**Fig. S1:**
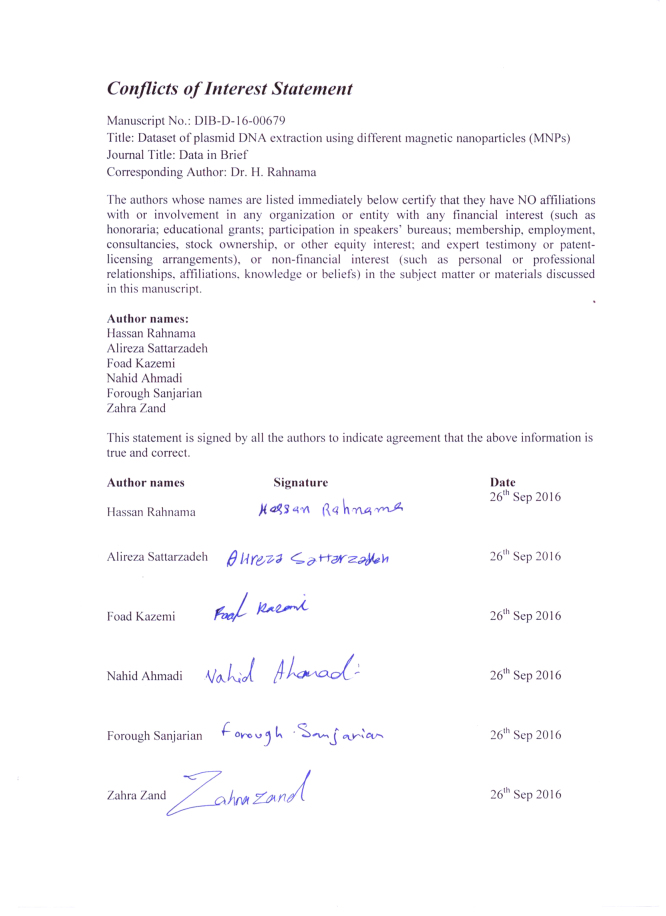


**Fig. 1:**
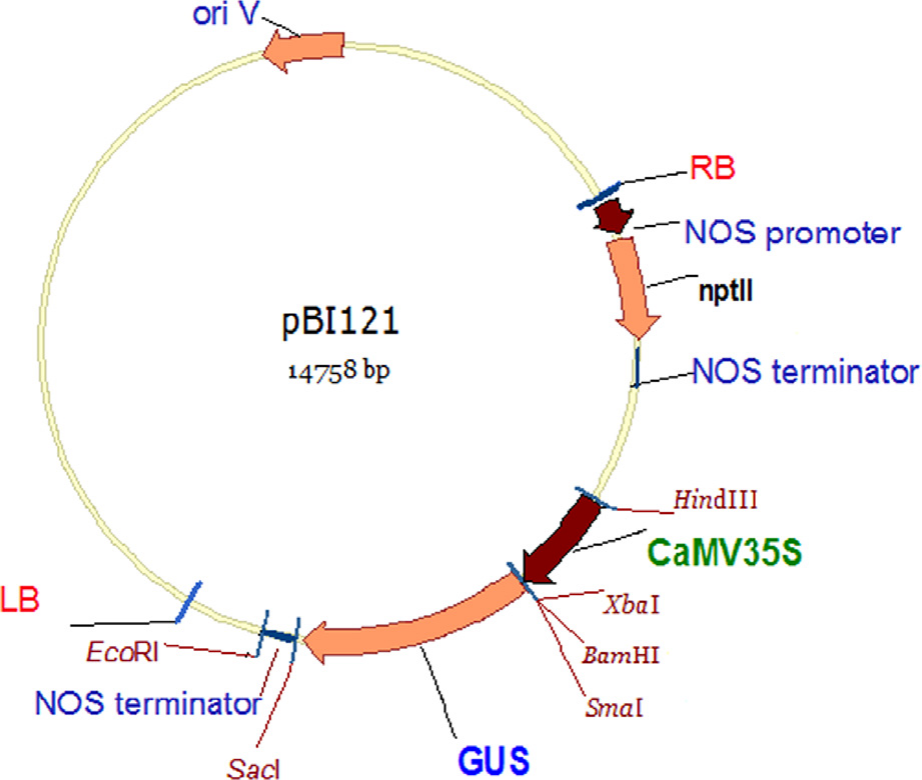
Physical structure of pBI121 plasmid. RB, Right border; LB: Left border; *npII*: neomycine phosphotransferase gene; CaMV35s: Cauliflower mosaic virus promoter; GUS: β-*glucuronidase* gene; *HindIII*, *XbaI*, *BamHI*, *SmaI*, *SacI*, *EcoRI*: restriction enzymes.

**Fig. 2:**
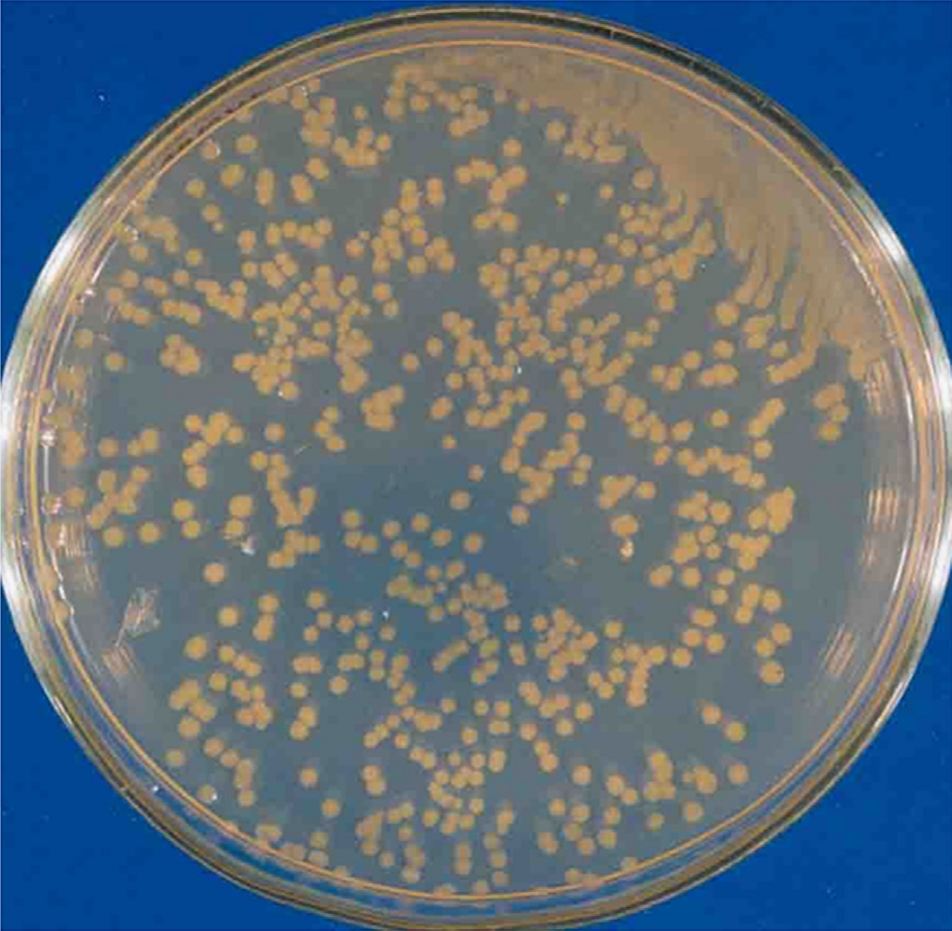
*E.coli* transformed using pBI121 plasmid. Bacterial colonies indicate the bacteria recipient kanamycine resistance through pBI121 plasmid.

**Fig. 3:**
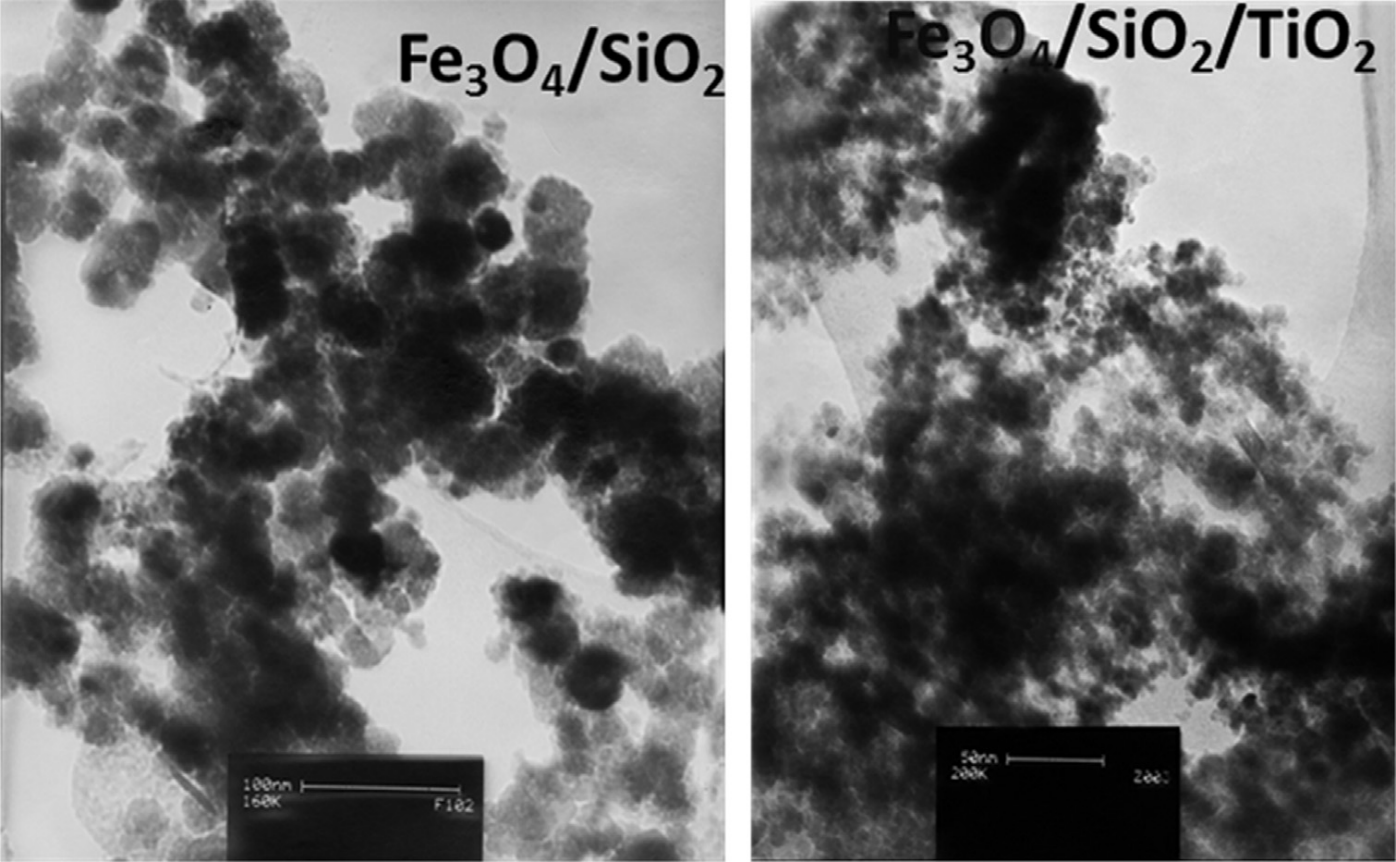
TEM micrographs of Fe_3_O_4_/SiO_2_ and Fe_3_O_4_/SiO_2_/TiO_2_.

**Fig. 4:**
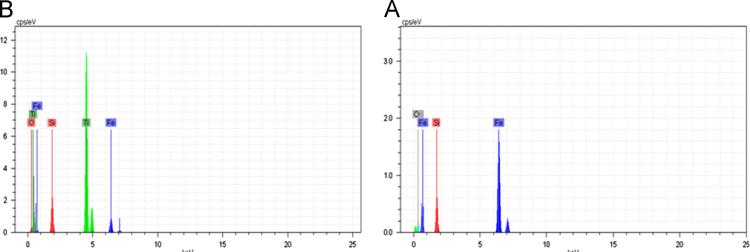
EDS data of Fe_3_O_4_/SiO_2_ (A) and Fe_3_O_4_/SiO_2_/TiO_2_ (B).

**Fig. 5:**
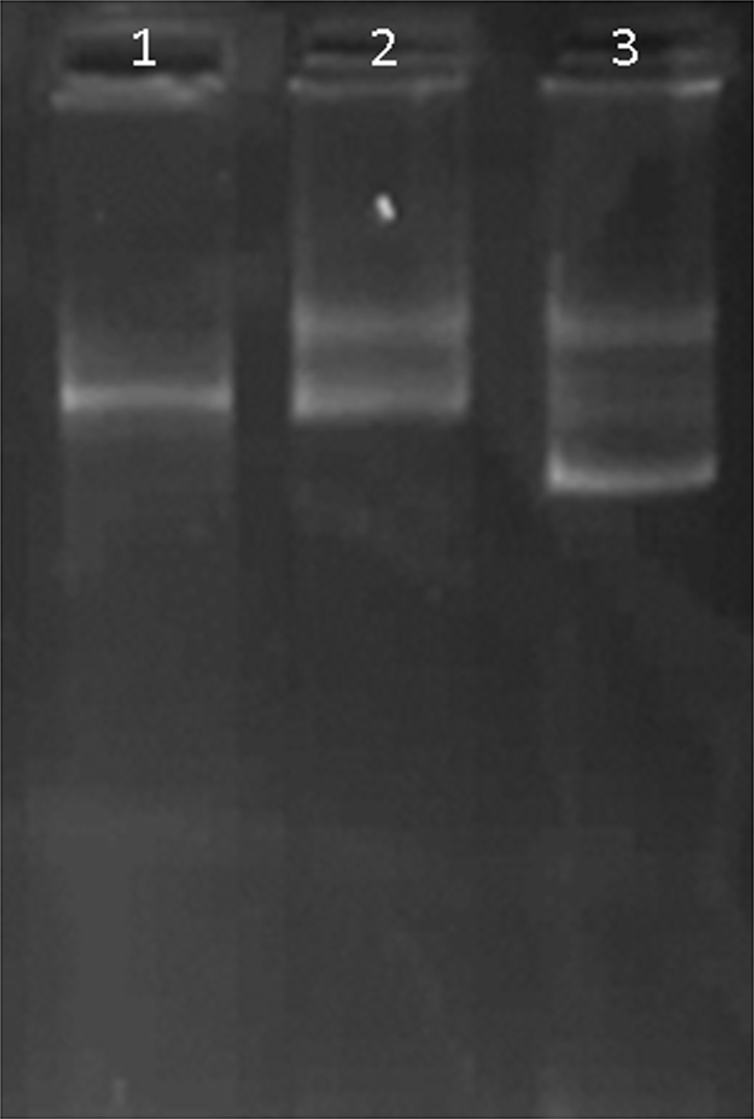
Plasmid DNA extraction using MNPs. 1- Fe_3_O_4_, 2- Fe_3_O_4_/SiO_2_/TiO_2_, 3- Fe_3_O_4_/SiO_2_.
